# Urbanisation-driven land degradation and socioeconomic challenges in peri-urban areas: Insights from Southern Europe

**DOI:** 10.1007/s13280-022-01701-7

**Published:** 2022-01-29

**Authors:** Samaneh Seifollahi-Aghmiuni, Zahra Kalantari, Gianluca Egidi, Luisa Gaburova, Luca Salvati

**Affiliations:** 1 Department of Physical Geography and Bolin Centre for Climate Research, Stockholm University, 106 91 Stockholm, Sweden; 2 Navarino Environmental Observatory, Costa Navarino, 24001 South-west Messenia, Greece; 3 Department of Sustainable Development, Environmental Science and Engineering (SEED), KTH Royal Institute of Technology, 100 44 Stockholm, Sweden; 4 Department of Agricultural and Forestry Sciences (DAFNE), University of Tuscia, Via S. Camillo de Lellis, 01100 Viterbo, Italy; 5 Department of Economics and Law, University of Macerata, Via Armaroli 43, 62100 Macerata, Italy

**Keywords:** Climate, Land fragmentation, Soil, Southern Europe, Urbanisation

## Abstract

Climate change and landscape transformation have led to rapid expansion of peri-urban areas globally, representing new ‘laboratories’ for the study of human–nature relationships aiming at land degradation management. This paper contributes to the debate on human-driven land degradation processes by highlighting how natural and socioeconomic forces trigger soil depletion and environmental degradation in peri-urban areas. The aim was to classify and synthesise the interactions of urbanisation-driven factors with direct or indirect, on-site or off-site, and short-term or century-scale impacts on land degradation, focussing on Southern Europe as a paradigmatic case to address this issue. Assuming complex and multifaceted interactions among influencing factors, a relevant contribution to land degradation was shown to derive from socioeconomic drivers, the most important of which were population growth and urban sprawl. Viewing peri-urban areas as socio-environmental systems adapting to intense socioeconomic transformations, these factors were identified as forming complex environmental ‘syndromes’ driven by urbanisation. Based on this classification, we suggested three key measures to support future land management in Southern European peri-urban areas.

## Introduction

Land degradation reflects natural and/or human processes that negatively affect soil functionality and ecosystem services within local/regional ecological systems. Land degradation is intrinsically related to the sensitivity, resilience and carrying capacity of ecosystems, and to the vulnerability of local communities, with soil depletion increasing faster than, or at roughly the same rate as, built-up areas (Zorn and Komac 2016). As a key environmental threat, land degradation has already reached global critical thresholds (Gibbs and Salmon 2015; Ferreira et al. 2021a). Different forms of land degradation can affect the quality and productivity of soils, and limit various soil ecosystem services in a given environmental system. Sustainable management and mitigation of land degradation, particularly in areas where environmental vulnerability is already a challenge, can improve soil health and structure, and support food production and regional resilience to climate change and anthropogenic pressures (e.g. Colantoni et al. 2015).

In the United Nations Convention to Combat Desertification (UNCCD), ‘land degradation’ is defined as a reversible process of desertification in arid, semi-arid, and dry sub-humid regions, resulting from various factors, including climate variations and human activities (UNCCD 2019a). Land degradation results in a (more or less intense) diminution or destruction of the biological potential of land, and can ultimately lead to desertification through drastic processes of soil depletion. While land degradation as a natural phenomenon has always been a concern worldwide and primarily is addressed in rural areas, the scientific understanding of its causes and consequences, especially in peri-urban and urban areas, is still not well-developed, highlighting an existing knowledge gap in land and soil management.

Land degradation in Southern Europe, as a paradigmatic region of advancing economies, experiencing intense spatial disparities, local/regional climate change, accelerated land take and soil consumption (European Environment Agency—EEA 2019), is still framed in traditional debates on rural development, focussing on environmental issues in less resilient and usually not economically well-developed local communities. Soil depletion and land degradation risks are seen as complementary to economic and social marginalisation, associated with rural poverty in peripheral areas and extensive use of cropland as the primary source of income and wealth. UNCCD Annex IV (addressing desertification issues in Mediterranean countries) interprets land degradation as an issue associated globally with rural areas experiencing environmental challenges depending on a structural development deficit (UNCCD 2019b; Robinson et al. 2019). However, population growth in coastal areas, the evolution from land-saving to land-consuming settlements, widespread peri-urbanisation and rising human pressure in metropolitan regions of Southern Europe—as in many other parts of the world—suggest and justify completely different considerations, shifting the focus of the land degradation debate from strictly rural communities to more mixed/complex environments including peri-urban areas (Recanatesi et al. 2016).

Despite largely under-investigated, land degradation in peri-urban areas is intimately associated with spatial and temporal changes and interactions of natural and human drivers (Salvati and Zitti 2008). Semi-arid climate regimes and extreme weather events, urban expansion and unsustainable land management, spatial disparities, overgrazing, more frequent (and/or severe) wildfires, crop intensification, and declining soil quality have been demonstrated to accentuate land degradation sensitivity in peri-urban areas (Doblas Miranda et al. 2018; Egidi et al. 2020b; Halbac-Cotoara-Zamfir et al. 2020). These processes represent a complex interconnection between natural and socioeconomic drivers involved in fringe land degradation in Southern Europe (Duvernoy et al. 2018; Ferreira et al. 2021b).

Various processes of land degradation occur in Southern European peri-urban areas, involving multiple social (e.g. demography, population trends, and lifestyle), economic (e.g. industrialisation, tourism development, infrastructural expansion, urban sprawl), and environmental (e.g. local climate change) factors with their impacts being still relatively poorly understood (Borrelli et al. 2018; Zambon et al. 2018a; Robinson et al. 2019). The few recent research contributions addressing these issues have examined: (i) the importance of rural landscape transformation into dispersed settlements (e.g. Zambon et al. 2018b); (ii) loss of semi-natural vegetation in fringe areas (e.g. Ruiz and Sanz-Sánchez 2020); (iii) the increasing risk of soil salinisation due to increasing groundwater abstractions as a result of concurrent residential and agricultural land/water uses (e.g. Tomaz et al. 2020); (iv) the processes involved in soil erosion and contamination (e.g. Ferreira et al. 2021a); and (v) the role of urban sprawl in increasing land degradation sensitivity (e.g. Salvati et al. 2012). However, it is also critically important to investigate the causal chains among influencing drivers and processes triggering land degradation in peri-urban areas (Recanatesi et al. 2016).

Peri-urban areas represent new 'laboratories' for the study of the intrinsic human–nature relationship to specify mitigation and adaptation strategies for land management, likely different than those already applied in rural areas. As a scientific contribution, in this paper we summarise and synthesise the key land degradation drivers and processes derived by urbanisation in Southern European peri-urban areas. Relevant factors are thus classified with direct or indirect, on-site or off-site, and short-term or century-scale impacts on land degradation, and their interactions with land degradation processes are assessed.

To clarify the role of interacting natural and socioeconomic factors that contribute to land degradation in peri-urban areas, we adopt the ‘syndrome’ concept (Smiraglia et al. 2016) for associated environmental challenges, representing a complex ensemble of factors acting synergistically and determining various land degradation processes (Bajocco et al. 2018). Based on empirical findings derived from earlier studies, this paper contributes to stimulating the current literature and the development of relevant management strategies for land degradation in Southern European peri-urban areas, by interpreting/classifying the main natural and socioeconomic factors affecting land degradation in these areas.

We synthesised and assessed impacts of drivers and processes of land degradation derived by urbanisation into seven classes of environmental challenges, with (i) direct or indirect, (ii) on-site or off-site and (iii) short-term or long-term contributions to land degradation. This synthesis provides a basis for investigating spatial and temporal changes in land sensitivity to degradation in peri-urban areas, and identifies pivotal factors whose impact can be generalised to broader spatial scales (including the whole Mediterranean region, the rest of Europe and other similar socioeconomic contexts all over the world). These findings also contribute to design more appropriate management strategies for degradation mitigation and adaptation to associated socioeconomic changes. Based on the empirical results of the analysis, we formulated some suggestions for research, policy, and planning measures for sustainable land management in Southern Europe.

Focussing on peri-urban areas, our study highlights the need for management strategies fundamentally dependent on the environmental, socioeconomic, institutional, and historical background of concerned regions. More generally, our study goes beyond the normative dictates of the UNCCD convention by initiating a specific debate on socio-environmental dynamics in peri-urban areas experiencing rapid changes worldwide, that were previously not well-studied regarding their land degradation, but potentially more vulnerable than rural areas.

## Urban expansion into Southern European fringe land

Southern European countries, located around the Mediterranean Sea, experienced a twofold increase in their population between 1970 and 2010 and, based on projections, they will be global urban hotspots with the highest percentage of urban land by 2030 (García-Nieto et al. 2018). For a long time, rapid population growth and large-scale internal migration from rural to urban areas were the main drivers of urbanisation in this region (Sudhira et al. 2004). In more recent times, with current ageing and negative natural population growth rates (i.e. number of deaths exceeding number of births), international immigration and rural-to-urban migration looking for better economic opportunities, and sub-urbanisation of local populations searching for larger, more comfortable and close-to-nature dwellings have fuelled urban expansion in Southern European countries (Egidi et al. 2020a). Consequently, urbanisation is expanding faster than population growth in recent times (Salvati 2019). Major examples of these trends can be seen in cities such as Porto and Lisbon in Portugal, Barcelona, Madrid, Valencia, and Seville in Spain, Marseilles and the Rhone valley in France, Milan, Bologna, Rome, Naples, and the Veneto region in Italy, as well as Athens and Thessaloniki in Greece (EEA 2006, 2019).

Urban expansion in Southern Europe has taken the form of disperse settlements in peri-urban areas as well as compact and dense metropolitan areas. Urbanisation often occupies agricultural land and potentially leads to local/regional environmental changes, such as intense droughts and desertification (Iosifides and Politidis 2005). These conditions contribute to a rural exodus or development of infrastructures in fragile coastal areas for tourism and housing development purposes. Such changes result in environmental degradation and socioeconomic challenges (Orgiazzi et al. 2016).

A refined understanding of changes in land-use patterns, socioeconomic processes and the consequent environmental implications, is required to support adaptive regional planning to the urgent social demands raised in Southern Europe during recent years. Most of the cities in Southern Europe are shifting towards polycentrism associated with less compact forms of settlement, which leads to spatially discontinuous urban expansion. The consequent land-use changes trigger various land degradation processes in fringe areas (Upadhyay et al. 2006).

## Classification of land degradation drivers and processes derived by urbanisation in Southern European peri-urban areas

Land degradation in rural northern parts of the Southern Europe is mainly associated with the inherent abandonment of agricultural activities, deterioration of landscape structures, unsustainable management of natural resources (i.e. soil), and uncontrolled socioeconomic development in terms of poverty, human pressure, rural-to-urban migration, and settlement densification (Halbac-Cotoara-Zamfir et al. 2020). However, multiple and often more complex drivers can be responsible for land degradation in peri-urban areas in Southern Europe, as in other ‘urban’ parts of the world. These drivers are mainly associated with socioeconomic factors, including demography, human land uptake, resource extraction, poverty, and land fragmentation (Iosifides and Politidis 2005; Salvati and Zitti 2007; Abu Hammad and Tumeizi 2012).

Urbanisation—as a major socioeconomic process within the Southern European region—involves and interacts with various environmental processes and contributes both directly and indirectly to regional land degradation. In this respect, land degradation is driven by a wide spectrum of interacting forces, associated with both short-term/intensive and century-scale processes. These forcing factors lead to complex environmental syndromes that require dedicated management and policy strategies for mitigation and adaptation (Halbac-Cotoara-Zamfir et al. 2020). To provide a complete overview of the most common environmental syndromes related to land degradation in peri-urban areas of Southern Europe, we classified land degradation drivers and processes derived by urbanisation based on three distinctive criteria: (i) impact characteristic (direct vs indirect), (ii) place-specificity/spatial scale (on-site vs off-site, i.e. narrow scale or broader scale), and (iii) temporal scale (short-term/intensive vs century-scale effects). These drivers and processes are synthesised through a holistic literature review and the proposed classification is presented in Table 1 with their specific impacts discussed in section “Impacts of land degradation drivers and processes derived by urbanisation in Southern Europe” of this paper for Southern European peri-urban areas.

**Table 1:** Land degradation drivers and processes derived by urbanisation in Southern Europe, classified based on impact characteristic (in rows), spatial scale (in columns), and temporal scale (with stars: ***long-term/century-scale impacts; **medium-term/decadal impacts; *short-term impacts)

Impact characteristic	Spatial scale		
	On-site	Mixed	Off-site
Direct	Population growth, urban expansion, and soil sealing*	Urban growth, competition for water, and soil salinisation**	Rising human pressure, vegetation cover degradation, and soil pollution**
Indirect	Urban sprawl/sub-urbanisation, social changes, and biodiversity loss***	Intensive use of fringe cropland, less availability of grazing lands for a relatively stable mass of livestock resulting in overgrazing on vacant land, and soil compaction**	Socioeconomic development, rural exodus (rural-to-urban migration), rural land abandonment, and soil erosion/landslides***
	Climate change and urban heating***		

## Impacts of land degradation drivers and processes derived by urbanisation in Southern Europe

### Population growth, urban expansion, and soil sealing

Although urban expansion is inherently influenced by social development and economic structure, population growth is a major driver of urbanisation. Urban expansion drivers are becoming dramatically endogenous in Southern Europe, fed by internal redistribution, inter-urban migration, and rural exodus (EEA 2006). Coastal regions have experienced demographic concentration and expansion of urban infrastructures and built environments to supply the demands of an increasing population (Rabehi et al. 2019). 'Littoralisation'—i.e. the concentration of population and economic activities in coastal regions—is thus a considerable feature of urbanisation in Southern Europe that is intensified in recent years thanks to late industrialisation, rising income and wealth due to continuous growth in international tourism. The globalising economy and associated de-structuring of traditional rural societies have also contributed to urban expansion into coastal districts (Salvati et al. 2018).

Mediterranean urban areas, especially in Southern Europe, have been identified as examples of massive population growth and uncontrolled urbanisation—at least between the early 1950s and the early 1990s (Benassi et al. 2020). Recent compact urban forms in Southern Europe (e.g. Athens in Greece and Barcelona in Spain) mainly represent the legacy of accelerated demographic growth in recent past (Di Feliciantonio et al. 2018; Duvernoy et al. 2018; Salvati et al. 2019). While population growth in the Northern Mediterranean region has slowed down since the 2000s, it is accelerating in the Southern parts as a result of continuous rural-to-urban migration flows.

Demographic data provided by the United Nations Statistical Office (UN 2021) indicate that, between 1950 and 2000, the population of the five largest Southern European countries (Portugal, Spain, France, Italy, and Greece) doubled. From 2000 to 2050, the population is projected to decline in Southern and South-Eastern European countries (Bosnia and Herzegovina, Bulgaria, Croatia, Montenegro, Romania, Serbia and the former Yugoslav Republic of Macedonia except Albania), slightly increase in Cyprus, and significantly increase in North African and Eastern Mediterranean countries (UN 2012). Demographic changes lead to local and regional land-use changes, characterised by expansion of urban and peri-urban areas and (mainly agricultural) land abandonment in the surrounding areas (Barbero-Sierra et al. 2013).

‘Unmanaged’ land consumption (i.e. rapid conversion from natural forest, agricultural land and rangeland to built-up areas) is a direct consequence of urban expansion (Mundia and Anya 2006; Zambon et al. 2018a), contributing to soil sealing through expansion of impervious surfaces (Ferreira et al. 2018; Anker et al. 2019; de Albuquerque et al. 2020). Soil sealing is a drastic form of land depletion which is increasing in Mediterranean Europe (Kalantari et al. 2017), representing land degradation over a short period of time (Benassi et al. 2020). In coastal and lowland areas, soil sealing is also increasing due to seasonal tourism concentration (Freire et al. 2009) and consolidation of city networks (Salvati et al. 2016a). The degree of soil sealing can be graded depending on the percentage of the area covered by impermeable materials (Tobias et al. 2018). As such, the amount of per capita impervious land in a certain area can be used as a simple index to measure soil sealing trends over time.

### Urban growth, competition for water, and soil salinisation

Urban–rural disparities are weakened and replaced by an indistinct metropolitan continuum. This is a significant element reflecting the existence and relevance of medium-term land degradation processes in peri-urban areas. In a context of intense competition for natural resources, the impact of mixed landscapes and habitat fragmentation on land degradation should be addressed in a multi-disciplinary perspective, evaluating the intrinsic, double-sided role of spatial planning in both regulating and enabling degradation processes. Fragmentation of agricultural spaces in a context of massive spill-over of settlements onto fertile soils and the subsequent creation of patchy, relict vacant land with negative impacts on biodiversity and ecosystem services is an important dimension of environmental degradation in peri-urban areas (Salvati 2019).

Unsustainable use of water resources, a characteristic of Southern European coastal areas experiencing massive urbanisation and seasonal tourism loads, has led to a strong increase in water withdrawal and consumption that is affecting local and regional water resources from both quantitative and qualitative perspectives. While agriculture is undoubtedly an economic activity using most of the available water resources, especially through unsustainable irrigation practices, domestic, civil and industrial water consumptions have also risen substantially, increasing the pressure on groundwater resources (Mazi et al. 2014). A decline in groundwater level in coastal areas can result in marine water intrusion into fresh coastal groundwater, which causes salinisation of vulnerable soils when used for agricultural irrigation. Groundwater level decline may also exacerbate landslide risks in affected regions.

### Rising human pressure, degradation of vegetation cover, and soil pollution

In cases of unsustainable planning, urbanisation as an increased human pressure on land can reduce vegetation cover and green spaces, and increase the exposure of soil to various degrading drivers such as natural disasters and urban contaminants. Vegetation cover in peri-urban areas consists of: (i) native local species originally present in the area; (ii) native regional species originally absent from the urban area, but newly introduced to the area due to urbanisation disturbances; and (iii) alien species introduced by humans, establishing wild populations in urban environments. These vegetation types are affected by multiple factors of change including habitat transformation and fragmentation, modifications of micro-environmental conditions; and diversified human preference for certain species in urban environments compared with rural contexts (William et al. 2009). The combined pressure of these factors can lead to non-random gain or loss of vegetation cover, changes in species abundances and alteration of plant functional traits and the phylogenetic distribution of species.

Earlier studies have shown that land degradation and vegetation cover are strictly related in a cause-effect chain (Riva et al. 2017). Rising human pressure degrades vegetation cover, reduces density and diversity of plants, and alters species composition, limiting the capacity of nature over a medium period of time to supply important ecosystem services such as air filtering, temperature modification, wind and noise reduction, carbon sequestration, water storage, and drainage (Mexia et al. 2018). Off-site effects such as altered chemical composition of air, soil and water, and disturbances in vegetation communities through trampling, accelerated erosion, dumping of rubbish, garden clippings, and building rubble were also documented, together with anthropogenic removal of organisms or components of ecosystems (Williams et al. 2005).

Previous studies of land degradation in peri-urban areas have identified soil pollution, e.g. elevated concentrations of trace elements (e.g. Zhu et al., 2019), polycyclic aromatic hydrocarbons (e.g. Nicola et al., 2015) and antibiotics (e.g. Zhao et al., 2020), as a primary threat to peri-urban environments. Soil pollution by heavy metals is also a serious problem in many parts of the world, including the Southern European environments, and is mainly caused by atmospheric fallout from various sources, the most important being industrial and traffic emissions in urbanised areas (Bou Kheir et al. 2014). As such, the concentration of metal contamination in soils, including aluminium, chromium, iron, lithium, nickel, manganese, lead, and zinc, is measured significantly higher in urbanised landscapes than agricultural areas (Imperato et al. 2003; Cicchella et al. 2008). Proximity of industries to central urban areas may also create excessive pollution hazards for soils in case of accidents such as fire, explosion and oil spills.

Heavy metals are non-biodegradable and their biological elimination takes a long time. Soil pollution limits the ability of peri-urban areas to provide clean/healthy food and agroecosystem services (Huang et al. 2018; Yu et al. 2018), with significant effects on human health in the long-term (Cicchella et al., 2005). Therefore, soils with higher contamination concentrations should not be used for vegetable production, as the uptake of pollutants by crops entails a public health risk (Zhu et al. 2019; Li et al. 2020). Peri-urban areas with soil pollution would need agricultural restructuring and land consolidation to avoid further environmental damage.

### Urban sprawl/sub-urbanisation, social changes, and biodiversity loss

Social and lifestyle changes have leveraged a preference for sprawled settlements as the basis of latent sub-urbanisation patterns in most Southern European countries. This is intimately associated with increased use of natural resources (Zambon et al. 2018a). Urban sprawl leads to horizontal rather than vertical growth, demanding a large proportion of natural resources at the expense of farming and forest areas, semi-natural environments, and wetlands, and making landscapes more vulnerable to degradation in long-term through various disturbances such as biodiversity loss. Impacts of discontinuous sub-urbanisation patterns and processes on regional sustainability and socioeconomic resilience have been documented in many studies focussing on the intimate relationship between spatial configurations of human settlements and background conditions in metropolitan regions, including economic performance, and social issues (e.g. Gavalas et al. 2014; Pili et al. 2017; García-Nieto et al. 2018; Egidi et al. 2020a). Settlement dispersion driven by sub-urbanisation attenuates the process of over-densification in central districts and homogenises urban environments and landscapes (Salvati and Zitti 2008), which in turn increases vulnerability to multiple natural hazards (e.g. floods and wildfires) through social change and biodiversity loss (Zambon et al. 2018a).

### Local climate change and urban heating

Climate change is significantly influencing land degradation in long-term in Southern Europe (Salvati et al. 2016b). This is a major concern, as the probability and intensity of, e.g. hydro-climatic disasters are projected to increase due to climate change impacts (Kalantari et al. 2017). Urban areas usually display different climate conditions compared with the surrounding natural environment (Santamouris et al. 2011). Cities are characterised by intense built environment, industrialisation, and massive transportation of goods and people, all contributing to local heating, greenhouse gas emissions, and ultimately regional warming (Mohajerani et al. 2017; Martinelli et al. 2020). Although these issues are not specifically generated by urban sprawl, mitigating associated risks and planning for adaptation are demonstrated sometimes to be more complicated in cases of uncontrolled urban expansion which increases sensitivity to land degradation (Egidi et al. 2020b).

As a consequence of future climate change, extreme weather events, such as riverine and coastal floods, may occur more frequently in peri-urban settlements (e.g. Anker et al. 2019). Urban areas may also experience high temperatures due to the well-known ‘urban heat island’ (UHI) effect (e.g. Martinelli et al. 2020). The UHI effect has multiple causes, but the most relevant are the percentage of *albedo* expressed by urban surfaces, the thermal capacity of urban land and infrastructures, the conformation and orientation of buildings in relation to the direction and speed of wind, and the reduction of evaporating surfaces in urban areas (Mohajerani et al. 2017). The UHI effect creates a dome of heat, usually 150–200 m high, especially in winter and at night, and results in temperature inversion at high altitudes (Salvati et al. 2019). It also increases the surface temperature in urbanised areas and can affect soil chemical processes and the composition of macro- and micro-organism communities involved in land degradation (Ferreira et al. 2018). These processes are enhanced by urban growth and sprawl and can significantly affect the local climate, causing a shift towards higher aridity and further soil degradation.

Urban heating therefore creates significant environmental problems and strongly affects urban ecosystems (Yang et al. 2015). Wildfires are one of the most evident consequence of climate warming in peri-urban districts. Fires at the urban-wildland interface are becoming increasingly common in Southern Europe, as consequences of global and local climate change (higher temperatures, stronger winds, less soil/air humidity), biomass accumulation and poor management of fringe land, increased human pressure, and land speculation (Badia et al. 2011; Moreira et al. 2011; Mancini et al. 2018). Wildfires destroy biodiversity, fragment landscapes, deteriorate local environments and threaten soils (Fares et al. 2017). At the same time, being intricately linked with the atmospheric system through the carbon and nitrogen cycles, peri-urban soils host communities of plants and soil microorganisms. Increased thermal stress on these soil organisms—directly driven by wildfires or indirectly driven by climate change—may accelerate biological degradation of soils (e.g. loss of decomposing organisms and microbes) and chemical processes (e.g. organic matter content) involved in land degradation (Brevik 2012).

### Intensive use of cropland, less availability of grazing lands for a relatively stable mass of livestock resulting in overgrazing on vacant land, and soil compaction

Urban expansion normally takes productive agricultural land and transforms it into built environments with sealed and compacted soils (Li et al. 2018). While land conversion into built environment provides the most immediate economic returns when competition for land is intense (Pavon et al. 2003; Atis 2006), farmers in urbanising areas have to use their remaining cropland more intensively through changing to more profitable crops, or shifting to operations that require less investment in infrastructure, in order to compete with more profitable (alternative) land uses (Salvati et al. 2012, 2019). Taken together, these processes further fragment former patterns of land use and land cover, reducing the amount and size of habitats, and associated wildlife, and the degree of connection between remaining patches (Barlow et al. 1998; Pili et al. 2019), making the land more sensitive to degradation over a medium period of time.

Corresponding to drastic changes in agricultural landscape along metropolitan fringes (Recanatesi et al. 2016), a general decline in extensive livestock farming on fringe land and its concentration on vacant land have negatively impacted the use of green open spaces in Southern Europe over time. The remaining livestock farms on fringe land are mainly concentrated on a few undeveloped, small and spatially fragmented patches, possibly triggering soil pollution and loss in meadow biodiversity because of overgrazing (Zambon et al. 2018b). In this perspective, urbanisation can indirectly drive overgrazing when the surface area of grassland and meadows reduces because of land take and livestock amount, especially flocks of goats and sheep—typical of Southern European areas, remain stable. This happens mainly when few specialised farms resist to urbanisation pressures in fringe land, or when new livestock farms are established on vacant land to supply the increased human demands in urban and peri-urban areas (Zambon et al. 2018a).

### Socioeconomic development, rural exodus, rural land abandonment, and soil erosion

Human activities have significantly amplified land degradation process in Southern Europe over the past 50 years. Living standards for the rural population are partly determined by access to natural resources such as high soil and water quality, which are severely affected by urban expansion and associated desertification in long-term (Abu Hammad and Tumeizi, 2012). Industrialisation processes lead to rural land abandonment because of increased cultivation costs, decreased profits, and changes in trade regulations among countries (e.g. Atis 2006). Local job market in rural areas, especially those traditionally linked to the primary sector (i.e. agriculture), are potentially sensitive to changes in land availability and crop production due to land degradation (Barbier 2000), while employment in the new job market in peri-urban and urban areas, mainly within the tourism and hospitality sectors, may also be negatively affected by long-term impacts of land degradation in the forms of aridity and environmental degradation (Harte 2007).

Rural exodus is likely the most evident example of off-site effects of large-scale urban expansion and socioeconomic development on land degradation (Borrelli et al. 2018). Resulting depopulation and rural land abandonment cause soil erosion and increase landslide risks (Marignani et al. 2017). Land abandonment in turn determines biomass accumulation in forests and natural areas, possibly enhancing wildfire frequency and intensity (Barbero-Sierra et al. 2013). In this perspective, land vulnerability to natural disasters such as floods and windstorms, and associated environmental degradation, can significantly increase (Kefalas et al. 2019). Additional off-site disturbances associated with urbanisation of natural environments are demonstrated to increase the risk of natural disasters, such as floods, through more concentrated and larger volume of surface runoff (e.g. Kalantari et al. 2017; Ferreira et al. 2020), decrease inherent land capabilities to mitigate the negative impacts of such disasters (e.g. García-Nieto et al. 2018), and increase soil sensitivity and vulnerability to erosion in affected rural areas (e.g. Rodrigo-Comino et al. 2018).

## Discussion

Land degradation is one of the most serious effects of urbanisation in Southern Europe, involving multiple and diversified natural and socioeconomic drivers and processes. Among all, the most important factors include population growth and tourism expansion, industrial activities, climate change, increased land vulnerability due to lack of soil organic matter and disturbances in soil biological processes, over-exploitation of groundwater resources, overgrazing of vacant land, and triggering of natural disasters such as floods and wildfires (Loumou et al. 2000).

The present study revealed that the major issues deriving from the classified environmental syndromes are mostly related to spatially unbalanced natural resources, economic polarisation, social disparities, and poor land management. This creates a need to rethink socioeconomic models and planning practices, delineating a new balance between equity, social cohesion, economic competitiveness, and environmental security (Iosifides and Politidis 2005). Based on our synthesis, the following research, policy and planning measures are suggested for sustainable land management in Southern European peri-urban areas.

### Broadening the focus from ‘strictly rural’ to ‘peri-urban’ regions in land degradation assessment and policy

So far, policies contrasting land degradation have been especially designed for, and applied to, rural areas (Salvati et al. 2012). Land degradation hotspots (e.g. ‘critical’ land where degradation processes are active or highly probable in the short term) have mainly been delineated in strictly rural areas according to defined population density thresholds (Zambon et al. 2018b). However, land degradation hotspots can also develop in medium-size and disperse urban areas (Salvati et al. 2018). The unrested expansion of metropolitan regions in Southern Europe requires that both theoretical and operational perspectives in land degradation assessment and management will be focussed on ‘peri-urban’ regions, broadening a traditional vision of land degradation as a ‘strictly rural’ phenomenon (Salvati and Zitti 2008).

In recent decades, many Southern European cities have pursued a scattered spatial setting approach, promoting sub-centres, strengthening metropolitan poles, stimulating economic activities in fringe areas, and re-locating urban functions (Egidi et al. 2020a; Ronsivalle 2020). Although polycentric growth is on the ‘normative agenda’ in European regional policy (Davoudi 2003), the social and productive characteristics of urban areas differ strongly across Europe from North to South (Salvati et al. 2018). These differences make polycentric development a driver (instead of a solution) of urban sprawl and associated land degradation processes, which should be contrasted with specific measures developed for a broader spatial scale than only rural areas (Recanatesi et al. 2016). To support such measures, future research and assessments of land degradation in Southern Europe should enlarge their spatial perspective to encompass peri-urban areas (Zambon et al. 2018b).

### The role of environmental assessment in informing dedicated policy strategies

Since the social, economic and environmental processes involved in land degradation vary between countries, territorial heterogeneity affects the ‘vulnerability’ status of peri-urban land (Zambon et al. 2018a). A high-resolution, diachronic assessment of the (rising) level of land sensitivity to degradation in Southern European peri-urban areas is thus particularly meaningful, and cannot be simply derived from the standard methodologies applied in rural districts (e.g. the Environmentally Sensitive Area Index developed by Salvati and Zitti (2007), among others). A specific assessment for peri-urban areas can reflect the peculiar characteristics of urban-wildland interfaces, focussing on the most frequent/common land degradation forms in metropolitan districts (e.g. soil pollution/contamination, erosion), while considering some issues that also occur in rural areas (soil compaction and salinisation).

Novel exploratory and interpretative approaches are especially required to investigate the intimate relationship between environmental degradation at metropolitan fringes and social phenomena such as ethnic and class segregation, social filtering and gentrification, population ageing and shrinkage, which can be regarded as latent engines of peri-urban expansion (Gavalas et al. 2014; Pili et al. 2017; Di Feliciantonio et al. 2018; Duvernoy et al. 2018). The progressive mismatch between the expanding urbanised areas and an increasing resident population is a possible result of this interaction. Thus optimal regulation requires continuous updating of planning tools and local development policies at the most appropriate spatial scales of intervention (Salvati et al. 2016b).

With this perspective in mind, our synthesis indicates that the intrinsic mismatch between population growth and settlement expansion can be used as a possible early-warning indicator of urban system change towards unsustainability and land degradation (Benassi et al. 2020). Finely tuned policies for mitigating land degradation in metropolitan regions definitely require more advanced and science-based knowledge of the drivers of urban change (Diaz-Pacheco and Garcia-Palomares 2014), specific monitoring approaches, and planning rules adapted to largely transformative peri-urban environments.

### Sustainable planning strategies for peri-urban hotspots of land degradation in Southern Europe

Based on the classification of environmental syndromes made in this study, land degradation hotspots (i.e. areas with high susceptibility/vulnerability to land degradation drivers and processes) should be identified with precise boundaries in peri-urban regions as sustainable planning targets (Woltjer 2014). In these areas, specific mitigation measures for land degradation should be designed in a context of sustainable urban development and reduction of ecological, social, and economic disparities (Metternicht et al. 2019). One priority is to use the soil/land with low agricultural productivity in metropolitan fringes for more profitable purposes (i.e. urbanisation and industrialization), since using those lands for agricultural activities would expose farmers to changing (mainly declining) yields due to soil characteristics and local climate change impacts in peri-urban areas.

Planning practices to promote sustainable conservation of coastlines, wetlands, and farmland, and to integrate tourism with agriculture should be strengthened throughout Southern Europe. However, attempts by the Mediterranean Commission on Sustainable Development and other affected countries to produce strategic proposals (e.g. through the National Action Plan to combat desertification), and the willingness of regional governments to intervene at local level still seem to be restricted.

A comprehensive planning and management strategy should be promoted, based on specific land zoning that distinguishes between different land-use types in terms of landscape disturbances (Salvati and Zitti 2007). Three major classes can generally be identified in metropolitan regions: (i) built-up areas; (ii) seriously compromised areas with patches of rural land adjacent to urban areas that are affected by soil degradation; and (iii) moderately compromised areas with plots partly under cultivation and other land that could be used for temporary agriculture and recreational activities. This would allow specific measures to be adapted for land degradation in metropolitan regions. Policies based on the degree of disturbance of each land unit could contribute to rationalisation of land conservation, land degradation mitigation, and desertification risk reduction in metropolitan fringes (Abu Hammad and Tumeizi 2012).

In this perspective, our classification of environmental syndromes based on their impact characteristics, and spatial and temporal scales, can be particularly useful for designing dedicated planning strategies in diversified territorial contexts. Spatial heterogeneity and site-specific processes of change can impede the design of effective mitigation and adaptation strategies for land degradation, negating national efforts/guidelines and often requiring a bottom-up strategy. Such planning approaches would need participation of local stakeholders, and improved coordination of (environmental) protection actions at multiple governance levels.

Policies could be specifically adapted to the site-specific environmental spirals and calibrated according to their various dimensions considered in our classification. This approach could produce an overview of adaptive measures addressing the specificity of each territory. While the environmental syndromes highlighted and discussed in this study can be considered to provide a generalised or, in some cases, a partial overview of land degradation drivers and processes in Southern European peri-urban areas, they represent a comprehensive set of problem aspects that regional planning strategies and policies should target, instead of only addressing a single land degradation dimension (e.g. individual driver or unique process of degradation). Actions responding to multiple (and complex) environmental syndromes should be developed to achieve the land degradation neutral target in Southern European peri-urban areas and in other parts of the world (European Court of Auditors, 2018).

## Concluding remarks

While urbanisation has been closely related to economic development and demographic change, heterogeneous patterns and processes of regional growth and change reflect the uneven distribution of urbanisation, the subtle impact of demographic dynamics and the consequent implications for land resource management and environmental sustainability. Differences in patterns of metropolitan growth and change in a paradigmatic region such as Southern Europe reflect regional divides in socio-demographic, economic and environmental variables. Reconnecting impacts of regional-scale socioeconomic change with local-scale environmental dynamics definitely contributes to an enriched knowledge of socioeconomic histories and the consequent environmental degradation processes, outlining how a study of differences under assumptions of non-linearity and complex system thinking is key to understand future socio-environmental trends in Southern Europe.

In this line of thinking, our study shows the inherent complexity of the socioeconomic phenomena underlying sprawl dynamics and (more or less) uncontrolled expansion of metropolitan areas in Southern Europe. Because of these interlinked phenomena, integrated approaches are required to assess the intrinsic loss of natural resources (e.g. water, soil, air) in both physical and monetary terms in relation to the inherent socio-demographic change associated with land transformations. Environmental accounting approaches capable of linking land-use changes in socially diversified peri-urban areas with the main aspects of environmental degradation and with the regional economic transformations of local society can be particularly important in this regard.

The current study contributes to broader debates on socio-environmental dynamics in peri-urban areas experiencing rapid changes worldwide, that were previously not well-studied regarding their land degradation, but potentially more vulnerable than rural areas. The adoption of the environmental ‘syndrome’ concept has also supported the collective interpretation of natural and socioeconomic factors with corresponding land degradation processes and resulting environmental challenges. This study also provides key research, policy and planning measures for land degradation management in Southern European peri-urban areas.

The multiple linkages between urban sprawl, climate change, population dynamics, landscape transformations, and land degradation risk also require in-depth comparative assessments. With this perspective in mind, we specifically encourage studies within a multi-disciplinary arena, stimulating further literature aimed at discussing these deserving issues (desertification, urbanisation, socio-demographic dynamics)—proposing new theoretical frameworks, with empirical approaches, comparative works and case studies that will provide the necessary, informed ground to science and policy.
